# Ising-type Magnetic Anisotropy in CePd_2_As_2_

**DOI:** 10.1038/s41598-017-07595-w

**Published:** 2017-08-04

**Authors:** M. O. Ajeesh, T. Shang, W. B. Jiang, W. Xie, R. D. dos Reis, M. Smidman, C. Geibel, H. Q. Yuan, M. Nicklas

**Affiliations:** 1 Max Planck Institute for Chemical Physics of Solids, Nöthnitzer Str. 40, 01187 Dresden, Germany; 2 Center for Correlated Matter and Department of Physics, Zhejiang University, Hangzhou, 310058 China

## Abstract

We investigated the anisotropic magnetic properties of CePd_2_As_2_ by magnetic, thermal and electrical transport studies. X-ray diffraction confirmed the tetragonal ThCr_2_Si_2_-type structure and the high-quality of the single crystals. Magnetisation and magnetic susceptibility data taken along the different crystallographic directions evidence a huge crystalline electric field (CEF) induced Ising-type magneto-crystalline anisotropy with a large *c*-axis moment and a small in-plane moment at low temperature. A detailed CEF analysis based on the magnetic susceptibility data indicates an almost pure |±5/2〉 CEF ground-state doublet with the dominantly |±3/2〉 and the |±1/2〉 doublets at 290 K and 330 K, respectively. At low temperature, we observe a uniaxial antiferromagnetic (AFM) transition at *T*_*N*_ = 14.7 K with the crystallographic *c*-direction being the magnetic easy-axis. The magnetic entropy gain up to *T*_*N*_ reaches almost *R* ln 2 indicating localised 4 *f*-electron magnetism without significant Kondo-type interactions. Below *T*_*N*_, the application of a magnetic field along the *c*-axis induces a metamagnetic transition from the AFM to a field-polarised phase at *μ*_0_ *H*_*c*0_ = 0.95 T, exhibiting a text-book example of a spin-flip transition as anticipated for an Ising-type AFM.

## Introduction

Materials crystallising in the ThCr_2_Si_2_-type structure comprise of such prominent compounds as the first heavy-fermion superconductor CeCu_2_Si_2_^1^ and BaFe_2_As_2_, a parent compound to the iron-based high-temperature superconductors^2^, making them especially attractive for solid-state research in the past decades. The discovery of heavy-fermion superconductivity in CeCu_2_Si_2_ resulted in extensive studies which became crucial for the understanding of unconventional superconductivity. In Ce-based heavy-fermion systems the strength of the hybridisation between the Ce-4*f* electrons and the conduction electrons is particularly important for the physical behaviour at low temperatures. There, the competition between Kondo effect and Ruderman-Kittel-Kasuya-Yoshida (RKKY) interaction along with crystalline electric field (CEF) effects lead to a large variety of different ground-state properties, which might be tuned using external control parameters such as chemical substitution, magnetic field and hydrostatic pressure^3–7^.

A large number of the Ce-based compounds crystallising in the ThCr_2_Si_2_-type structure order antiferromagnetically at low temperatures. Their magnetism is commonly determined by a large magneto-crystalline anisotropy. This leads to the presence of distinct field-induced metamagnetic transitions^8–15^. Depending on the strength of the magnetic anisotropy, the nature of the metamagnetic transition(s) may differ. Additionally, the spin structure in the antiferromagnetic (AFM) phase plays an important role in the field-induced metamagnetic transition(s). In this regard, CePd_2_As_2_ offers the opportunity to study magnetism in a localised moment antiferromagnet with a huge magneto-crystalline anisotropy.

Recently, the physical properties of polycrystalline CePd_2_As_2_, which crystallises in the ThCr_2_Si_2_-type structure, were reported^16^. CePd_2_As_2_ undergoes an antiferromagnetic (AFM) ordering at *T*_*N*_ ≈ 15 K and shows evidence of a metamagnetic transition. However, a detailed investigation on single crystalline samples is necessary in order to understand the magnetic properties. In this work, we report on the magnetic anisotropy of single crystalline CePd_2_As_2_. To this end, we carried out magnetic susceptibility, magnetisation, electrical-transport and specific-heat measurements. Our results reveal an Ising-type magnetic anisotropy which accounts for a text-book-like spin-flip metamagnetic transition. The CEF level scheme could be fully resolved based on our experimental data. Furthermore, our analysis suggests a simple, collinear A-type antiferromagnetic spin structure in the AFM state.

## Results

### Magnetic susceptibility and heat capacity

**Figure 1:**
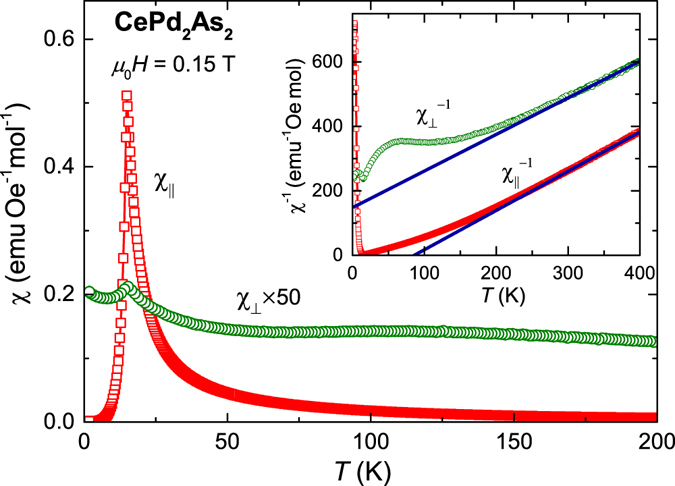
Temperature dependence of the magnetic susceptibility *χ* = *M*/*H* for magnetic fields applied parallel (${\chi }_{\parallel }$) and perpendicular (*χ*_⊥_) to the *c*-axis. Here, *M* is the magnetisation and *H* is the magnetic field. The inset displays the inverse magnetic susceptibility as a function of temperature. The solid lines represent fits of a Curie-Weiss law, *χ*(*T*) = *C*/(*T* − *θ*_W_), to ${\chi }_{\parallel }(T)$ and *χ*_⊥_(*T*) in the temperature interval 300 K ≤ *T* ≤ 400 K and 350 K ≤ *T* ≤ 400 *K*, respectively.

The temperature dependence of magnetic susceptibility *χ* of CePd_2_As_2_ with magnetic fields applied parallel (${\chi }_{\parallel }$) and perpendicular (*χ*_⊥_) to the crystallographic *c*-axis are depicted in Fig. 1. *χ*(*T*) shows a sharp peak at *T*_*N*_ = 14.7 K for both field orientations indicating the AFM transition, in good agreement with the results previously reported on polycrystalline samples^16^. Remarkably, ${\chi }_{\parallel }$ is two orders of magnitude larger than *χ*_⊥_ implying the presence of a strong magnetic anisotropy. The inverse magnetic susceptibilities ${\chi }_{\parallel }^{-1}(T)$ and ${\chi }_{\perp }^{-1}(T)$ are plotted in the inset of Fig. 1. Above room temperature, the susceptibility data can be fit by a Curie-Weiss law, *χ*(*T*) = *C*/(*T* − *θ*_W_), where *C* and *θ*_W_ are the Curie constant and the Weiss temperature, respectively. We find ${\theta }_{{\rm{W}}}^{\parallel }=86\,K$ and *μ*_eff_ = 2.56 *μ*_*B*_ for $H\parallel c$ and ${\theta }_{{\rm{W}}}^{\perp }=-130\,K$ and *μ*_eff_ = 2.65 *μ*_*B*_ for *H* ⊥ *c*. The obtained effective moments are slightly enhanced compared with the calculated value of 2.54 *μ*_*B*_ for a free Ce^3+^ ion. The deviation of ${\chi }_{\parallel ,\perp }(T)$ from a Curie-Weiss law below room temperature can be attributed to CEF effects.

**Figure 2:**
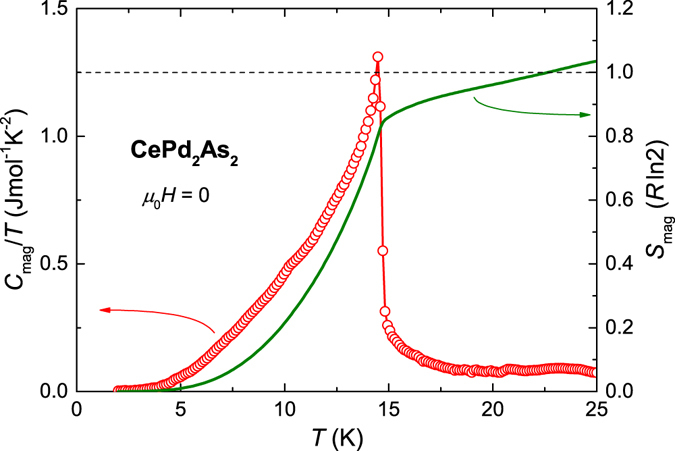
Temperature dependence of the magnetic contribution to the specific heat *C*_mag_ of CePd_2_As_2_, plotted as *C*_mag_(*T*)/*T* (left axis). *C*_mag_ was estimated by subtracting the specific heat of the non-magnetic reference compound LaPd_2_As_2_ from that of CePd_2_As_2_. The calculated magnetic entropy *S*_mag_(*T*) is displayed in the unit of *R*ln2 (right axis).

For a free Ce^3+^ ion with total angular momentum *J* = 5/2, the ground state consists of 6–fold degenerate levels. In the presence of a CEF with a tetragonal symmetry, these degenerate levels split into three doublets which are energetically separated from each other. The physical properties of CePd_2_As_2_ are greatly influenced by the relative thermal population of these energy levels. In CePd_2_As_2_, the magnetic contribution to the entropy, estimated from the specific heat data, reaches ~85% of *R* ln 2 at *T*_*N*_ (see Fig. 2). This indicates that the ground state is a doublet well-separated from the excited CEF levels and that the Kondo effect is rather weak. Further evidence for the localised character of the Ce moments comes from the magnetisation data discussed below.

### Magnetisation

**Figure 3:**
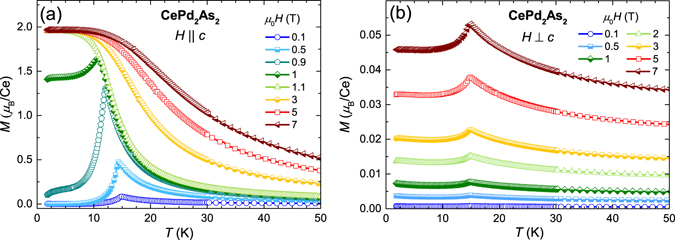
Temperature dependence of the magnetisation *M*(*T*) measured under various magnetic fields applied (**a**) parallel and (**b**) perpendicular to the crystallographic *c*-axis.

Figure 3 presents the temperature dependence of the magnetisation measured under various magnetic fields applied parallel and perpendicular to the crystallographic *c*-axis. For $H\parallel c$, *T*_*N*_ shifts to lower temperatures upon increasing the magnetic field, which is expected for an antiferromagnet. As the magnetic field approaches 1 T, the peak in *M*(*T*) corresponding to the AFM transition disappears and a broad step-like feature with a saturation of *M*(*T*) toward low temperatures develops. However, for *H* ⊥ *c* the position of peak corresponding to *T*_*N*_ is independent of the magnetic field and still clearly visible at 7 T. These different behaviours reflect the large magnetic anisotropy present in CePd_2_As_2_. We note that, the sudden decrease in the magnetisation below *T*_*N*_ for $H\parallel c$ compared to that of *H* ⊥ *c* suggests that the crystallographic *c*-direction is the magnetic easy-axis.

**Figure 4:**
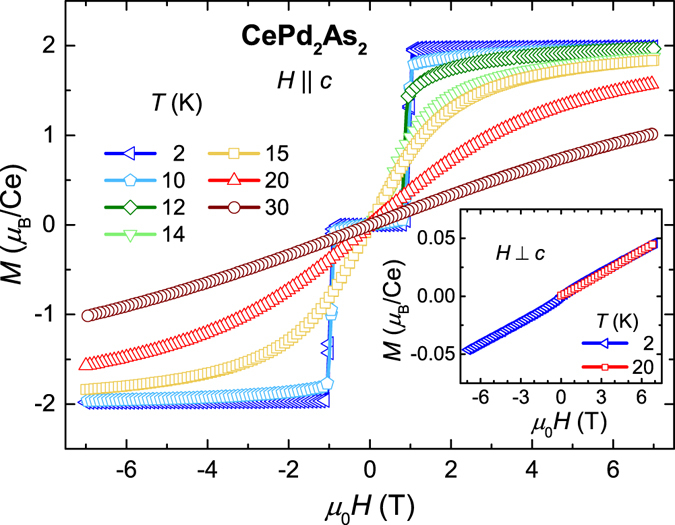
Isothermal magnetisation *M*(*H*) measured at different temperatures for magnetic fields applied along the *c*-axis and perpendicular to the *c*-axis (inset).

The isothermal magnetisation *M*(*H*) at 2 K, shown in Fig. 4, displays a sudden jump at *μ*_0_ *H*_*c*_ ≈ 1 T for $H\parallel c$ followed by an immediate saturation. The observed saturation moment of 2.0 *μ*_*B*_/Ce is in reasonable agreement with the theoretical value of *g*_*J*_ *J* = 2.14 *μ*_*B*_ (where *g*_*J*_ = 6/7) expected for a free Ce^3+^ ion. The sudden jump in magnetisation to the saturation value is a typical signature of a spin-flip metamagnetic transition. In the spin-flip process, the spins in the AFM sublattice, which are antiparallel to the field direction, are flipped at *H*_*c*_. Hence, the antiferromagnetism changes to a field-polarised phase in a sudden, single step. The sharp nature of the jump in magnetisation with a small hysteresis point to a first-order type transition. At higher temperatures, the metamagnetic transition in *M*(*H*) broadens and saturates at much higher fields. In the case of *H* ⊥ *c* (inset of Fig. 4), the magnetisation increases monotonously and reaches at 7 T only 2.5% of the saturation value for $H\parallel c$. Furthermore, magnetisation measurements in pulsed fields up to 60 T show a linear increase without any tendency to saturation (not shown). This suggests the absence of any metamagnetic transition for *H* ⊥ *c*, which stipulates the huge magnetic anisotropy in CePd_2_As_2_.

### Electrical transport

**Figure 5:**
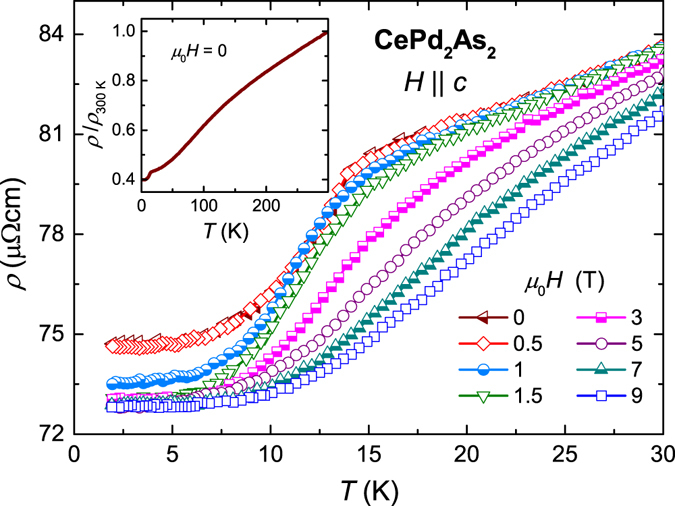
Temperature dependence of electrical resistivity *ρ*(*T*) of CePd_2_As_2_ measured at various magnetic fields applied along the *c*-axis. Inset: normalised resistivity *ρ*/*ρ*_300 *K*_ as a function of *T*.

The electrical resistivity *ρ*(*T*) of CePd_2_As_2_ upon cooling displays a metallic behaviour with a broad curvature at intermediate temperatures, before showing a pronounced kink at about 15 K indicating the AFM transition (inset of Fig. 5). The broad curvature in the resistivity may be due either to interband scattering or to weak additional spin scattering originating from thermal population of excited CEF levels. At low temperatures, the AFM ordering leads to a sudden decrease in *ρ*(*T*) due to the loss of spin-disorder scattering contribution below *T*_*N*_. Figure 5 shows the *ρ*(*T*) data recorded at different magnetic fields applied along the *c*-axis. Upon increasing the field up to 1 T, the kink indicating *T*_*N*_ shifts to lower temperatures and becomes washed out, in good agreement with the results from the magnetic susceptibility. Moreover, above 1 T the residual resistivity shows a sudden reduction which coincides with the metamagnetic critical field.

**Figure 6:**
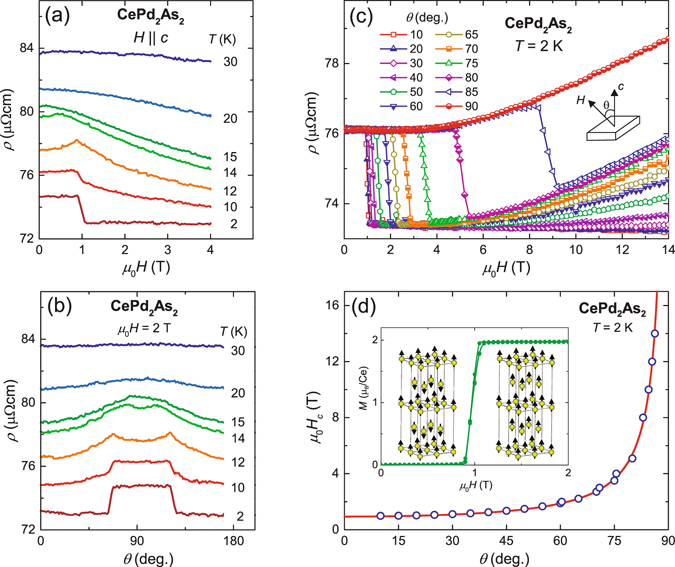
(**a**) Magnetic field dependence of *ρ* for field parallel to *c*-axis. (**b**) *ρ* as a function of the angle (*θ*) between the magnetic field *H* and *c*-axis at different temperatures. (**c**) Magnetic field dependence of *ρ* for different angles *θ* at *T* = 2 K. (**d**) Variation of metamagnetic critical field *H*_*c*_ as function of *θ* at *T* = 2 K. The solid red line is a fit to the data using the equation *H*_*c*_ = *H*_*c*0_/cos (*θ*). The inset illustrates the magnetic structures in the A-type antiferromagnetic and in the field-polarised phase.

The field and angular dependencies of the resistivity, plotted in Fig. 6(a–c), give further insights into the nature of the metamagnetic transition. At low temperatures, upon increasing the magnetic field *ρ*(*H*) suddenly drops at the onset of the metamagnetic transition at the critical field *μ*_0_ *H*_*c*_ ≈ 1 T. This feature can be attributed to a Fermi-surface reconstruction while going from the AFM to the field-polarised phase. At higher temperatures, the metamagnetic transition in *ρ*(*H*) broadens. A small increase in *ρ*(*H*) is observed just below *H*_*c*_ in the AFM phase for temperatures close to *T*_*N*_. This could be due to an increased scattering during the spin-flip process associated with the transition from the AFM to field-polarised state^17^. Above the AFM transition temperature, *ρ*(*H*) displays a gradual decrease upon increasing magnetic field, suggesting a crossover from the paramagnetic to the field-polarised phase. The variation of *ρ* as function of the angle (*θ*) between the magnetic field (*μ*_0_ *H* = 2 T) and the crystallographic *c*-axis at different temperatures is shown in Fig. 6(b). The step-like behaviour at lower temperatures changes to a gradual decrease in resistivity above *T*_*N*_, where the system undergoes a crossover from paramagnetic to the field-polarised phase. Above 30 K, the resistivity becomes independent of the field orientation. Finally, Fig. 6(c) presents the resistivity as a function of field for different angles *θ*. The metamagnetic critical field *H*_*c*_ increases upon increasing *θ* and diverges for *θ* → 90°. No drop in *ρ*(*H*) is observed up to 14 T for field perpendicular to the *c*-axis. This is consistent with our magnetisation experiments.

## Discussion

**Figure 7:**
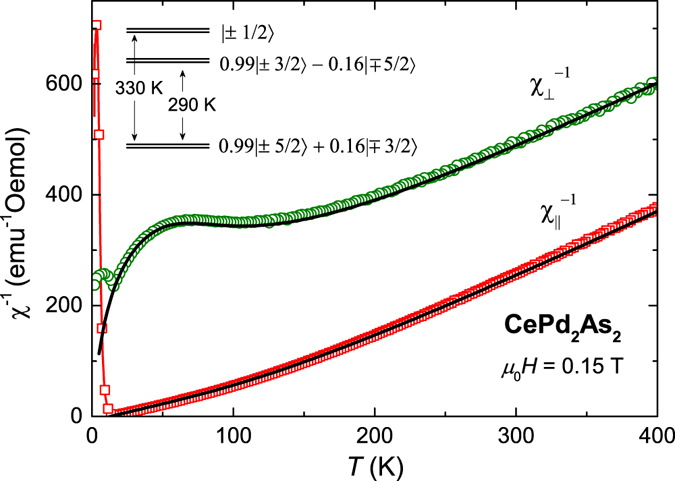
Temperature dependence of inverse magnetic susceptibility of CePd_2_As_2_. The solid lines are based on CEF calculation. A schematic representation of the CEF levels is shown in the inset.

In CePd_2_As_2_, the temperature dependence of the magnetic susceptibility below room temperature strongly deviates from a Curie-Weiss behaviour. This can be attributed to CEF effects. In order to establish the CEF scheme and learn more about the magnetic anisotropy in CePd_2_As_2_, we performed a detailed CEF analysis based on our magnetic susceptibility data. For a Ce atom in a tetragonal site symmetry, the CEF Hamiltonian can be written as,

1 $${ {\mathcal H} }_{{\rm{CEF}}}={B}_{2}^{0}{O}_{2}^{0}+{B}_{4}^{0}{O}_{4}^{0}+{B}_{4}^{4}{O}_{4}^{4},$$

where ${B}_{m}^{n}$ and ${O}_{m}^{n}$ are the CEF parameters and the Stevens operators, respectively^18, 19^. The magnetic susceptibility including the Van Vleck contribution is calculated as,

2 $${\chi }_{{\rm{CEF}},i}={N}_{A}{({g}_{J}{\mu }_{B})}^{2}\,\tfrac{1}{Z}\,(\sum _{m\ne n}2\,{|\langle m|{J}_{i}|n\rangle |}^{2}\,\tfrac{1-{e}^{-\beta ({E}_{n}-{E}_{m})}}{{E}_{n}-{E}_{m}}{e}^{-\beta {E}_{n}}+\sum _{n}\,{|\langle n|{J}_{i}|n\rangle |}^{2}\beta {e}^{-\beta {E}_{n}}),$$

where $Z={\sum }_{n}\,{e}^{-\beta {E}_{n}}$, *β* = 1/*k*_*B*_ *T* and *i* = *x*,*y*,*z*. The inverse magnetic susceptibility including the molecular field contribution *λ*_*i*_ is calculated as ${\chi }_{i}^{-1}={\chi }_{{\rm{CEF}},i}^{-1}-{\lambda }_{i}$. ${\chi }_{i}^{-1}(T)$ is fitted simultaneously to the experimental data for both field orientations (see Fig. 7). The data in the paramagnetic phase are well reproduced by the CEF model with a doublet ground state $|{{\rm{\Gamma }}}_{7}^{\mathrm{(1)}}\rangle =0.99|\pm \mathrm{5/2}\rangle +0.16|\mp \mathrm{3/2}\rangle$ and the excited doublet states $|{{\rm{\Gamma }}}_{7}^{\mathrm{(2)}}\rangle =0.99|\pm \mathrm{3/2}\rangle -0.16|\mp \mathrm{5/2}\rangle$ and $|{{\rm{\Gamma }}}_{6}\rangle =|\pm \mathrm{1/2}\rangle$ at 290 K and 330 K, respectively. An illustration of the CEF level scheme is shown in the inset of Fig. 7. The crystal field parameters extracted from the model are ${B}_{2}^{0}=-18.66\,{\rm{K}}$, ${B}_{4}^{0}=-0.22\,{\rm{K}}$ and $|{B}_{4}^{4}|=1.67\,{\rm{K}}$, with a molecular field contribution *λ*_*c*_ = −8 emu^−1^ mol along the *c*-axis. As pointed out by U. Walter^20^, the sign of the ${B}_{4}^{4}$ parameter cannot be determined from such kind of data, therefore only the absolute value is given. The *T*-dependence of the in-plane susceptibility (the hard direction) is very sensitive to the CEF excitation energies (*E*_1_, *E*_2_) and the admixture of $|\pm \mathrm{3/2}\rangle$ into the $|\pm \mathrm{5/2}\rangle$ state, and thus allows for a precise determination of these parameters. A doubling of the mean squared error in the fit of susceptibility is achieved by changing *E*_1_, *E*_2_ and the admixture coefficient from the optimum values by 3%, 4% and 5%, respectively. The crystal field parameters ${B}_{m}^{n}$ depends linearly on *E*_1_, *E*_2_ and the admixture coefficient. Therefore the uncertainty in the ${B}_{m}^{n}$ parameters are also estimated to be of the order of 5%. It is clear from our CEF analysis that the ground state is an almost pure $|\pm \mathrm{5/2}\rangle$ CEF doublet which is well-separated from the excited doublets. The saturation magnetisation along the *c*-axis for the obtained CEF ground state is 2.06 *μ*_B_/Ce, which is in good agreement with the experimental saturation magnetisation of 2.0 *μ*_*B*_/Ce. Furthermore, the CEF parameter ${B}_{2}^{0}$ is directly related to the paramagnetic Curie-Weiss temperatures ${\theta }_{{\rm{W}}}^{\perp }$ and ${\theta }_{{\rm{W}}}^{\parallel }$, along both principal crystallographic directions, as ${\theta }_{{\rm{CW}}}^{\perp }-{\theta }_{{\rm{CW}}}^{\parallel }=\frac{3}{10}{B}_{2}^{0}\mathrm{(2}J-\mathrm{1)}\,\mathrm{(2}J+\mathrm{1)}$ ^21, 22^. Using the experimental values of ${\theta }_{{\rm{W}}}^{\perp }$ and ${\theta }_{{\rm{W}}}^{\parallel }$, we obtain ${B}_{2}^{0}=-22.5\,K$ in good agreement to ${B}_{2}^{0}=-18.66\,{\rm{K}}$ from the CEF-model fit.

Deeper insights into the magnetic structure of the ordered phases in CePd_2_As_2_ can be obtained from the magnetisation and electrical resistivity data measured at different orientations of the magnetic field. The magnetisation data suggest that the crystallographic *c*-direction is the easy-axis of the magnetisation. Moreover, the small magnetisation in the *ab*-plane compared with the large magnetisation along the *c*-axis indicates an AFM structure with the spins pointing along the *c*-axis. In addition, the spins are locked along the *c*-axis by the magneto-crystalline anisotropy, as indicated by the absence of a metamagnetic transition for magnetic field up to 60 T applied perpendicular to the *c*-axis. These observations confirm that the moments in CePd_2_As_2_ are Ising-type. The Ising-nature of the spins is also supported by the angular dependence of the metamagnetic critical field extracted from the electrical resistivity data shown in Fig. 6(c). The resulting angular dependence of *H*_*c*_ is displayed in Fig. 6(d). *H*_*c*_(*θ*) increases sharply for *θ* → 90° and *H*_*c*_ is not detected for field oriented perpendicular to the *c*-axis.

In order to understand the angular dependence of *H*_*c*_(*θ*), we fit the data by the equation,

3 $${H}_{c}=\frac{{H}_{c0}}{\cos \,(\theta )}$$

where *H*_*c*0_ is the critical field for field parallel to the *c*-axis. Equation 3 describes the experimental data very well with *μ*_0_ *H*_*c*0_ = 0.95 T. In other words, the metamagnetic transition occurs only when the component of magnetic field along the *c*-axis reaches the value of *H*_*c*0_. Based on these results, we can conclude the following scenario for the spin structure of CePd_2_As_2_: in the AFM phase, the Ce moments are aligned along the *c*-axis and are locked along this axis by the magneto-crystalline anisotropy. When the component of external magnetic field along the *c*-axis exceeds *H*_*c*0_, the anti-parallel spins undergo a spin-flip transition to the field-polarised ferromagnetically ordered phase.

The single, sharp jump in the magnetisation with a weak hysteresis at the first-order metamagnetic transition from the AFM to the ferromagnetically polarised state points at a simple spin structure of the AFM phase. The small value of the critical field *H*_*c*0_, compared to the value of *T*_*N*_, indicates that, in terms of a Heisenberg model with a few different inter-site exchange interactions, the AFM ones are much weaker than the ferromagnetic (FM) ones. Because of the topology of the tetragonal body centered Ce sublattice, a strong FM interaction between atoms in adjacent layers competing with a weak in-plane AFM interaction would always result in a FM ground state. In contrast, a strong FM in-plane interaction with a weak AFM inter-plane interaction can easily account for all observations. In addition, we note that isovalent substitution of P for As results in a FM ground state^16^. Thus all these properties provide strong indication that the AFM structure of CePd_2_As_2_ is just a simple AFM stacking of FM layers. Substituting P for As is just turning the inter-plane exchange from weakly AFM to FM. Therefore, we propose a magnetic structure with weakly antiferromagnetically coupled FM layers of Ising-spins in the AFM state of CePd_2_As_2_, as illustrated in the inset of Fig. 6(d). A mean-field approximation based on a two-sublattice model can appropriately describe such a spin system. According to this model, the spin-flip occurs when the applied magnetic field is able to overcome the inter-layer AFM coupling. Therefore, the metamagnetic critical field can be expressed as *H*_*c*_ = *λ*_AFM_ *M*, where *λ*_AFM_ is the inter-sublattice molecular field constant and *M* is the magnetisation of the ferromagnetic state^23^. Similarly, the intra-sublattice molecular field constant *λ*_FM_ can be extracted from the relation ${T}_{N}=\frac{1}{2}C({\lambda }_{{\rm{FM}}}-{\lambda }_{{\rm{AFM}}})$, where *C* is the Curie constant $C={N}_{A}{\mu }_{0}{g}_{J}^{2}J(J+\mathrm{1)}{\mu }_{B}^{2}\mathrm{/3}{k}_{{\rm{B}}}$. By using the experimentally obtained values *H*_*c*0_, *M*_*S*_ and *T*_*N*_, the inter-layer AFM exchange strength (*z*_AFM_ *J*_AFM_) and intra-layer FM exchange strength (*z*_FM_ *J*_FM_) are calculated as −0.25 K and 9.83 K, respectively. Here, *z*_AFM_ and *z*_FM_ are the number of nearest-neighbour spins participating in the respective interactions. The large intra-layer FM exchange strength is consistent with the experimental observations and plays a crucial role in the first-order nature of the metamagnetic transition.

**Figure 8:**
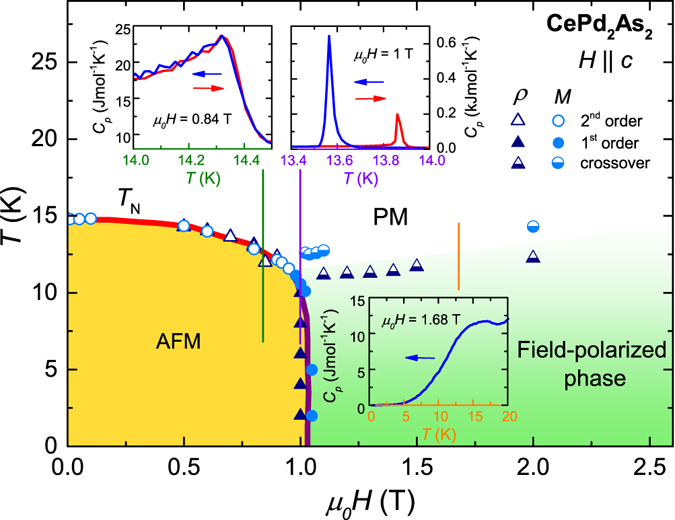
Magnetic phase diagram of CePd_2_As_2_ for $H\parallel c$ summarising the results from magnetisation and electrical resistivity data. The lines are guides to the eyes. The three insets present the temperature dependence of the specific heat at three representative magnetic fields as indicated by the vertical lines. The red and blue arrows in the insets indicate the direction of the temperature sweep.

The *T* − *H* phase diagram of CePd_2_As_2_ for $H\parallel c$, presented in Fig. 8, summarises our results. At low temperatures, application of a magnetic field induces a metamagnetic transition at *μ*_0_ *H*_*c*0_ = 0.95 T resulting in a field-polarised phase. Above *T*_*N*_, CePd_2_As_2_ shows a crossover behaviour from the paramagnetic to the field-polarised phase, reflected by the broad features in magnetisation and electrical resistivity. Additional information on the nature of the transitions between the various phases can be retrieved from specific-heat data. The temperature dependencies of the specific heat *C*_*p*_ of CePd_2_As_2_ for three representative magnetic fields are plotted in insets of Fig. 8. The specific heat was obtained upon heating and cooling by analysing the thermal-relaxation curves recorded in a standard relaxation-type measurement setup^24^. A cusp in *C*_*p*_(*T*) indicates the transition form the paramagnetic to AFM phase at 0.84 T. The second-order nature of this transition is evidenced by the absence of any thermal hysteresis between the data taken upon heating and cooling. In contrast, a strong thermal hysteresis and a spike-like anomaly in *C*_*p*_(*T*) at 1 T confirms the first-order nature of the metamagnetic transition from the AFM to the field-polarised phase. Finally, the crossover from the paramagnetic to the field-polarised phase at higher magnetic fields is reflected by a broad, hump-like feature in *C*_*p*_(*T*).

Because of its strong Ising anisotropy and simple magnetic behaviour, CePd_2_As_2_ is a nice example to illustrate a misinterpretation frequently encountered in the analysis and discussion of magnetic properties of Ce- and Yb-based compounds. The Weiss temperatures determined from Curie-Weiss fits to the high temperature part of the susceptibility are frequently argued to reflect the anisotropy, the sign and the magnitude of the exchange interactions, ignoring that in most cases they are dominated by the CEF effect. For example, such a misinterpretation occurred for the system Yb_2_Pt_2_Pb (see the discussion in A. Ochiai *et al*.^25^). In CePd_2_As_2_ this would lead to the conclusion that the exchange in the basal plane is strongly antiferromagnetic while the exchange along the *c*-direction is weaker and ferromagnetic. Our analysis clearly demonstrates that this conclusion would be completely wrong, because the Weiss temperatures determined from Curie-Weiss fits at high temperatures are dominated by the effect of the CEF. Except for special cases, CEF generally result in a seemingly AFM, negative *θ*_W_ for the direction of the small CEF ground-state moment and an apparently FM, positive *θ*_W_ for the direction of the large CEF ground-state moment.

We note that CePd_2_As_2_ presents quite some similarities, but also some instructive differences to the intensively studied system CeRu_2_(Ge_1−*x*_Si_*x*_)_2_. Upon increasing Si content, this system evolves from a FM ground state (with fully localised 4 *f* electrons) to an AFM ground state (for 0.4 < *x* < 0.935) and finally to a paramagnetic heavy fermion system^26, 27^. For all values of *x*, it presents a strong Ising character, with easy axis along the *c*-direction of the tetragonal ThCr_2_Si_2_-type structure. The CEF scheme has been precisely determined for *x* = 0, where the ground-state doublet is almost identical to that in CePd_2_As_2_, the mixing of $|\pm \mathrm{3/2}\rangle$ to the $|\pm \mathrm{5/2}\rangle$ states being slightly larger, 0.2 in CeRu_2_Ge_2_ compared to 0.16 in CePd_2_As_2_^28^. In the regime with an AFM ground state CeRu_2_(Ge_1−*x*_Si_*x*_)_2_ presents a pronounced metamagnetic transition at a small critical field, which increases from *μ*_0_ *H*_*c*_ = 0 at *x* = 0.4 to about 1 T at *x* = 0.93. Thus, at first glance, in this regime CeRu_2_(Ge_1−*x*_Si_*x*_)_2_ seems to be identical to CePd_2_As_2_. However, a closer inspection of the data show that for *H* < *H*_*c*_ the susceptibility in CeRu_2_(Ge_1−*x*_Si_*x*_)_2_ is at least one order of magnitude larger than in CePd_2_As_2_, i.e. the drop in *χ*(*T*) below *T*_*N*_ is at least one order of magnitude less pronounced^27, 29^. Furthermore, the phase diagram as a function of field (along the easy axis) is more complex, with evidences for up to 3 metamagnetic transitions^27^. Both differences can be attributed to a more complex AFM structure. In CeRu_2_(Ge_1−*x*_Si_*x*_)_2_ neutron scattering results evidence an incommensurate in-plane propagation vector, which makes it easier for the system to adapt its AFM configuration in a magnetic field in order to decrease its free energy^29^.

## Summary

We have investigated the magnetic properties and the CEF scheme of CePd_2_As_2_ by detailed temperature, magnetic field and angular dependent magnetic, thermodynamic and electrical transport studies on single crystalline samples. The detailed CEF analysis based on the magnetic-susceptibility data indicates an almost pure $|\pm \mathrm{5/2}\rangle$ CEF ground-state doublet with the dominantly $|\pm \mathrm{3/2}\rangle$ and the $|\pm \mathrm{1/2}\rangle$ doublets at 290 K and 330 K, respectively. CePd_2_As_2_ orders antiferromagnetically in a simple A-type order below *T*_*N*_ = 14.7 K. Our results imply a uniaxial AFM structure with spins locked along crystallographic *c*-axis. An external magnetic field applied along the *c*-axis induces a metamagnetic spin-flip transition at *μ*_0_ *H*_*c*0_ = 0.95 T leading to a ferromagnetic spin alignment. No metamagnetic transition is observed for a magnetic field perpendicular to the *c*-axis, proving the huge Ising-like anisotropy in CePd_2_As_2_.

## Methods

High quality single crystals of CePd_2_As_2_ have been synthesised by a self-flux method. CeAs precursors were prepared by heating a mixture of elemental Ce and As in a sealed evacuated quartz ampoule. Afterwards, CeAs precursors, Pd powder and As chips were weighed in a ratio of 1:2:1 and loaded into an alumina crucible, which was sealed in an evacuated quartz tube. The mixture was heated up to 950 °C for 5 days. The resulted powder was ground and cold pressed into a pellet, which was then heated up to 1160 °C, and held at this temperature for 24 hours, followed by slow cooling to 900 °C at a rate of 1.6 °C/h. Shiny plate-like single crystals of CePd_2_As_2_ were obtained directly upon cooling the furnace down to room temperature.

The crystal orientation and chemical homogeneity were checked by x-ray diffraction (XRD) and energy dispersive x-ray analysis (EDX), respectively. XRD measurements were carried out on a PANalytical X’pert MRD diffractometer with Cu *K*_*α*1_ radiation and a graphite monochromator. Magnetisation measurements were carried out in the temperature range 1.8 K–400 K and in magnetic field up to 7 T using a SQUID-VSM (MPMS3, Quantum Design). High-field magnetisation measurements up to 60 T in pulsed magnetic fields were performed at the Dresden High Magnetic Field Laboratory, Germany. The electrical transport experiments were carried out in the temperature range 2 K–300 K and magnetic fields up to 14 T using a Physical Property Measurement System (PPMS, Quantum Design). The electrical resistivity was measured using a standard four-terminal method, where electrical contacts to the sample were made using 25 *μ*m gold wires and silver paint. We applied an excitation current of 5 mA at a frequency of 87 Hz along crystallographic *a*-axis of the sample. The temperature dependence of specific heat was measured using the thermal relaxation method implemented in the PPMS. A thermal grease was utilised to ensure a good thermal coupling between the sample and the platform. In order to study the specific heat at the first-order phase transition, we analysed the slope of the time-dependent thermal relaxation curves following a method outlined by Lashley *et al*.^24^.

### Data Availability

The datasets generated during and/or analysed during the current study are available from the corresponding author on reasonable request.
